# A retinopathy in young patient with co-inheritance of heterozygous alpha + −thalassemia and sickle trait: a case report

**DOI:** 10.1186/s12886-017-0402-x

**Published:** 2017-01-18

**Authors:** Zohra Ouzzif, Aissam El Maataoui, Zeinab Traore, Asmae Biaz, Samira El Machtani, Abdellah Dami, Sanae Bouhsain, Nezha Messaoudi, Fatiha Benchrifa

**Affiliations:** 1 Biochemistry Department at Mohamed V Military Hospital, Rabat, Morocco; 2 Biochemistry Department, Faculty of Medicine And Pharmacy, Ibn Zohr University, Agadir, Morocco; 3 Heamatological Department at Mohamed V Military Hospital, Rabat, Morocco; 4 Private Ophthalmologists, Rabat, Morocco

**Keywords:** Sickle cells trait, Heterozygous alpha-thalassemia, Retinopathy, Neovascularization, Case report

## Abstract

**Background:**

The retinopathy is an uncommon complication in individuals with sickle cell trait except for the cases of sickle cell trait associated with systemic arterial hypertension, diabetes mellitus, syphilis, tuberculosis and sarcoidosis.

**Case Presentation:**

A retinopathy in a 16 year-old child with no history of consanguinity in the parents revealed a sickle S trait associated to heterozygous alpha thalassemia. His mother has Sickle cell anaemia (Hb SS) and his father is a carrier of heterozygous alpha-thalassemia status that it was unknown before.

**Conclusion:**

This case report describes a proliferative retinopathy in a 16 year-old patient with co-inheritance of heterozygous alpha + −thalassemia and sickle trait.

## Background

Sickle cell anaemia (SCA) is associated with life-threatening systemic manifestations results from homozygous inheritance of the haemoglobin (Hb) -S gene from both parents’ results in a homozygote (Hb SS). Also, SCA is due to compound heterozygosity for HbS and other haemoglobin variants like HbC, HbE, and HbD, or the many different genotypes of HbS-β thalassemia. People with SCA have abnormal haemoglobin which can distort red blood cells into a sickle shape. They break down more rapidly than normal red blood cells which can lead to deep anaemia with all his clinical manifestations [1]. The carrier individuals of sickle cell disease known as sickle cell trait (SCT) have one gene mutation resulting in the Hb AS genotype. although this is very rare SCT may have symptoms including splenic infarction at high altitude, with extreme exercise, or hypoxemia, isothenuria with loss of maximal renal concentrating ability, haematuria secondary to renal papillary necrosis, fatal exertional heat illness with exercise, sudden idiopathic death with exercise, glaucoma or recurrent hyphema following a first episode of hyphema, bacteruria or pyelonephritis associated with pregnancy, Renal medullary carcinoma in young people and early onset of end stage renal disease from autosomal dominant polycystic kidney [2]. Both heterozygous (−α/αα) and homozygous (−α/−α) α + thalassemia are associated with moderate reductions in both Mean Corpuscular Volume and Hb [3]. In SCA, homozygous α + thalassaemia inhibits polymerisation of HbS reducing sickling and the clinical manifestations of the disease [4]. In patients with sickle cell trait many cases of retinopathy has been reported [5, 6]. This paper describes the clinical, and the laboratory characteristics of a 16 year-old-patient with SCT associated to heterozygous α + thalassemia.

## Case Presentation

**Fig. 1:**
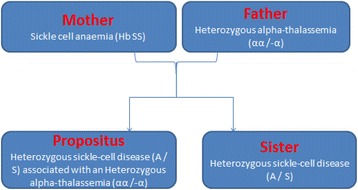
Family sickle cell pedigree chart

**Fig. 2:**
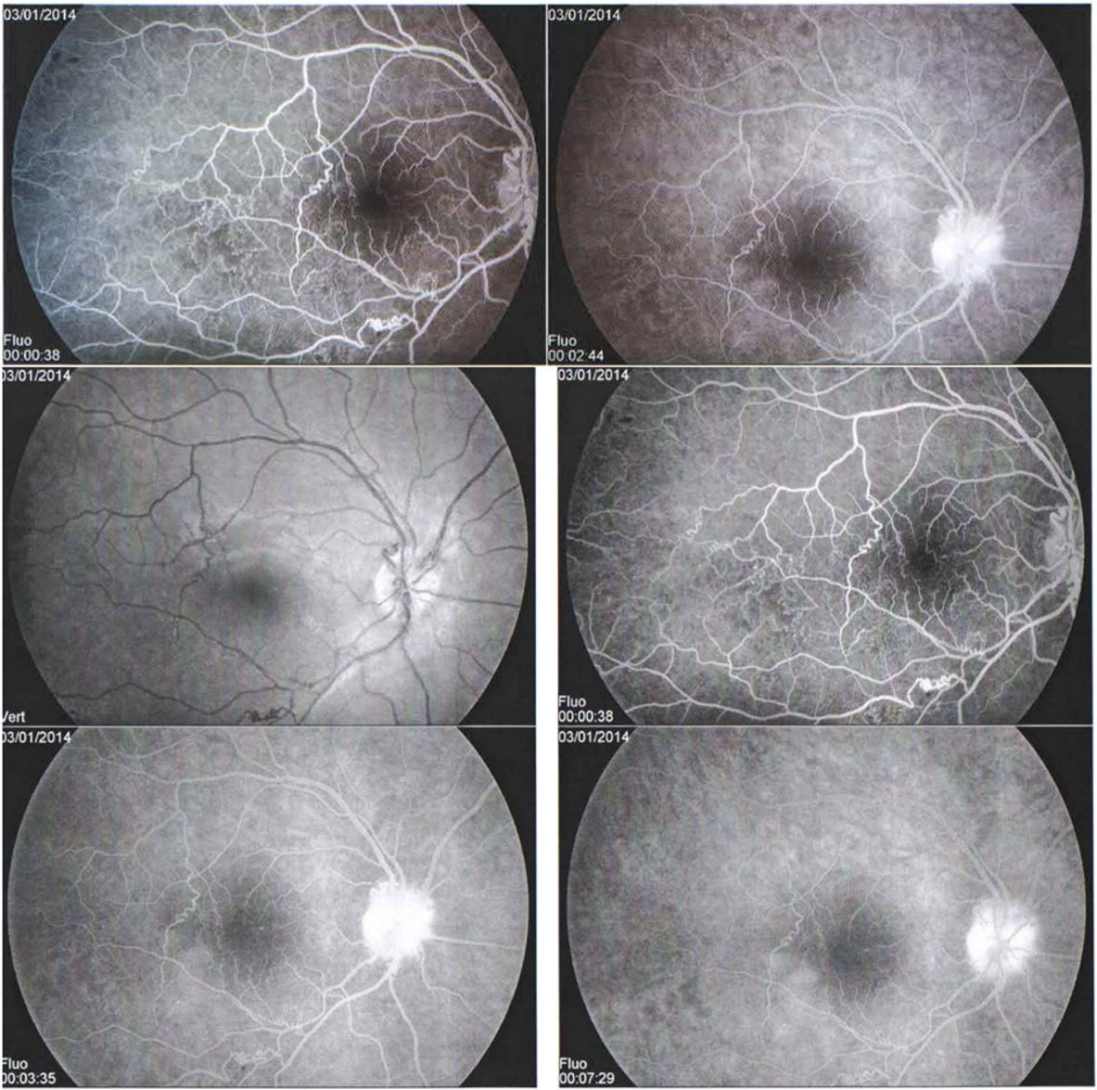
Fluorescein angiography of the patient’s right eye

**Fig. 3:**
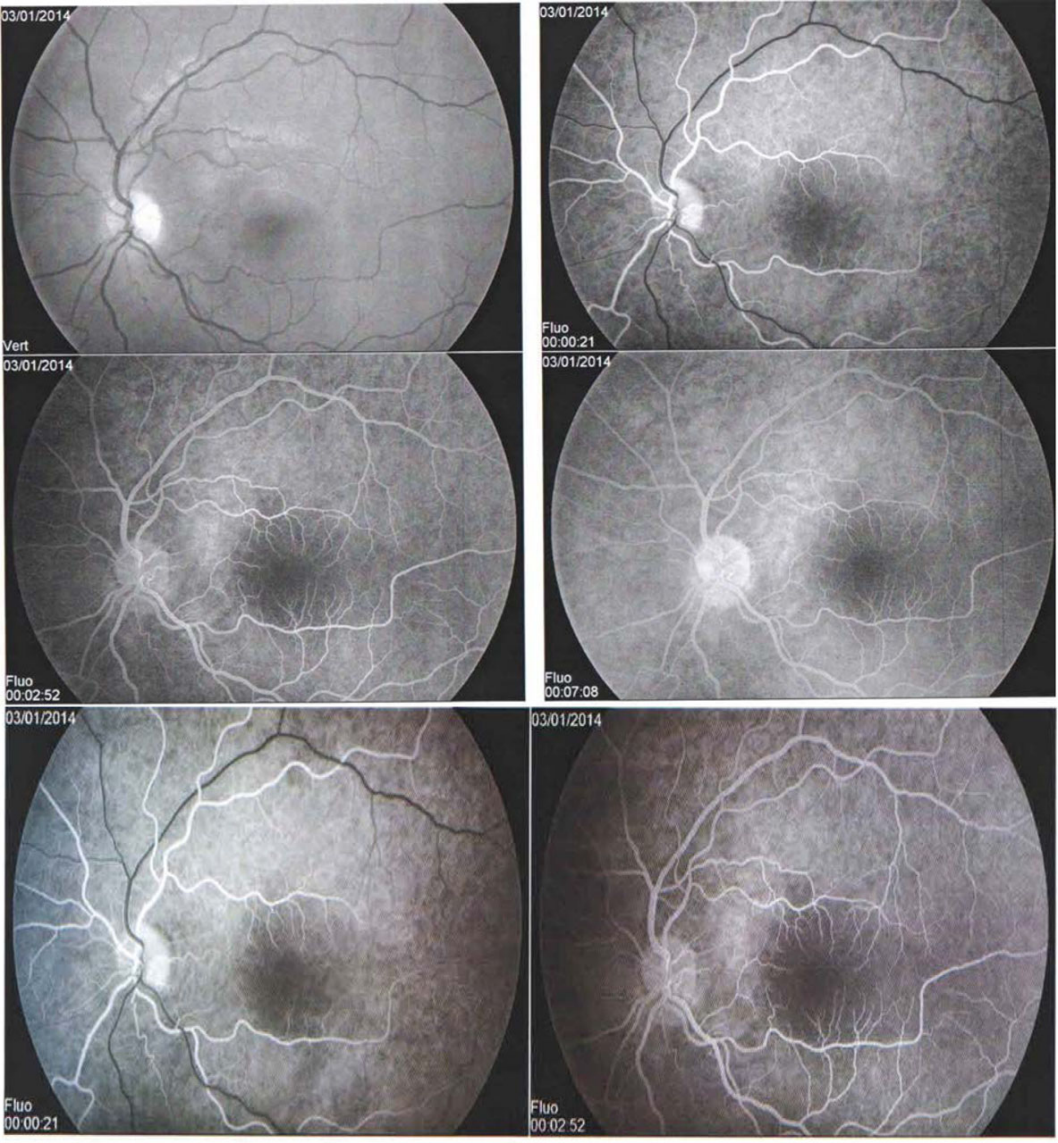
Fluorescein angiography of the patient’s left eye

A 16-year-old Caucasian patient with no known past medical history presented with 2 years of blurry vision in the right eye. He had no other ocular, medical, or surgical history. He rarely sought medical care and was on no medications. His mother has Sickle cell anaemia (Hb SS) (Fig. 1). In December 2013, he consulted an ophthalmologist for a history of sudden onset of amaurosis (Transient monocular visual loss) in the right eye associated with headaches and dizziness. Dilated fundus examination found unilateral papilledema in the left eye without loss of visual acuity. In the interpretation of Humphrey visual field testing, it has been reported that the right visual field showed some scotomas. The color vision examination was normal. Fundus fluorescein angiography (FFA) of the right eye revealed temporal capillary non-perfusion corresponding to retinal ischemia with no neovascularization (Fig. 2). FFA of the left eye showed venous tortuosity with no visible ischemic areas (Fig. 3). The retinopathy in the right eye was treated successively with scattered argon laser photocoagulation. There was no diagnosis of intracranial tumor, the magnetic resonance imaging and the brain computed tomography were normal.

The retinopathy and the fact that his mother has sickle cell anaemia motivated a screening test to identify variant and abnormal haemoglobins (electrophoresis, liquid high performance chromatography and polymerase chain reactions). A haemoglobin electrophoresis and genotyping revealed an heterozygous sickle-cell disease (A/S) associated with an heterozygous alpha-thalassemia (αα/-α), and the levels in percentage of the haemoglobin S (HbS), HbF, HbA and HbA2 was respectively 61.7, 0.8, 33.6 and 3.9% (Table 1). Patient’s haematological parameters showed normal haemoglobin level, hypochromia and microcytosis of the Blood red cells (RBC) (Table 1).

**Table 1:** The family biological parameters

Paramètres	Patient	Sister (A/S)	Father(αα/-α)	Mother (S/S)	Reference values
Hemoglobin (g/dl)	12.8	13.4	14.5	7.1	13–16
MCV (fl)	73.1	86.1	79.4	100.6	78–98
MCHC (pg)	22.91	27.4	24.1	32.7	25–35
Hématocrit (%)	40.2	42	47.7	21.9	37–49
RBC10^6^/μl	5.58	4.88	6.01	2.18	4.5–6.5
Ferritin (ng/ml)	14.5	27.9	28.8	553.2	11–336
Haptoglobin (g/l)	0.91	1.63	1.37	<0.072	
Total Bilirubin (mg/l)	5	8	4	23	3–12
Direct Bilirubine (mg/l)	<1	1.1	0.3	3.6	1–5
LDH (UI/l)	139	133	127	383	98–192
CRP (mg/l)	<1	2.5	<1	4.7	1–7.5
Hemoglobin A (%)	61.7	56	97.2	---	96–98
Hemoglobin S (%)	33.6	40.2		84.3	0
Hemoglobin A2 (%)	3.9	3.2	2.8	3	2–3
Hemoglobin F (%)	0.8	0.6		12.7	<2

*Abbreviations*: *MCV* mean corpuscular volume, *MCHC* mean corpuscular haemoglobin concentration, *RBC* red blood cell count, *LDH* lactate dehydrogenase

The other family members were studied after obtaining informed written consent. The screening was performed on family members because the patient has a sickle cell trait associated to an heterozygous alpha thalassemia and his mother has a sickle cell anaemia (Hb SS). The haematological parameters were normal for the patient’s father and sister even she has a sickle cell trait. Patient’s family genetic testing revealed the father’s alpha-thalassemia status which was unknown before (Table 1).

## Discussion and Conclusions

A retinopathy in a 16 year-old child with no history of consanguinity in the parents revealed a sickle S trait associated to heterozygous alpha thalassemia. His mother is a carrier of the sickle cell disease and his father is a carrier of heterozygous alpha-thalassemia status that it was unknown before. Sickle cell trait has no effect on haemoglobin concentrations [7], and heterozygous (−α/αα) α + thalassemia is associated with moderate reductions in both Mean Corpuscular Volume and haemoglobin concentration [3]. The retinopathy is an uncommon complication in individuals with sickle cell trait except for the cases of sickle cell trait associated with systemic arterial hypertension, diabetes mellitus, syphilis, tuberculosis and sarcoidosis [8–10]. But occurring more frequently in patients with the most clinically significant haemoglobinopathies: the SC, the S-thalassemia and the SS and after 20 year-old [9, 10]. The retinopathy is due to the vaso-occlusive processes. That is due to the red blood cells deformation or sickling, the result of polymerization of deoxyHbS and also high concentrations of unpolymerized oxidized HbS, modulated by cellular levels of HbF (foetal Hb), erythrocyte cation and water content, pH, temperature, and mechanical stresses that result in membrane damage and eventual failure. Sickling cells are red blood cells with abnormal shape and lower deformability which can cause them to undergo haemolysis (haemolytic anaemia) [1]. The haemolytic anaemia and the vaso-occlusive processes lead to retinal hypoxia, ischemia, infarction and neovascularization [9, 11]. This is due to their or be removed by macrophages in the spleen.

Homozygous alpha-thalassemia (α-/α-) inhibits in vivo sickling in SCD (homozygous sickle-cell disease). Indeed, Higgs et al. found that patients with SCD and homozygous alpha-thalassemia (α-/α-) had significantly higher red-cell counts and levels of haemoglobin and haemoglobin A2, as well as significantly lower haemoglobin F, mean corpuscular haemoglobin, mean corpuscular haemoglobin concentration, mean corpuscular volume, reticulocyte counts, irreversibly-sickled-cell counts, and serum total bilirubin levels, than those with SCA and normal alpha-globin-gene complement. Heterozygotes (α–/αα) had intermediate values between those of the patients with homozygous alpha-thalassemia (α-/α-) and normal alpha genes associated to SCD [12].

Fox et al. studied the influence of homozygous a + thalassaemia on the retinal complications in patients with homozygous sickle cell (SS) disease. homozygous a + thalassaemia reduces the extent of peripheral retinal vessel closure but has no apparent effect on the frequency of proliferative sickle retinopathy [13].

This case report describes a proliferative retinopathy in a 16 year-old patient with co-inheritance of heterozygous alpha + −thalassemia and sickle trait. Perhaps this co-inheritance may reduce the extent of peripheral retinal vessel closure. More studies with a big number of patients are needed for the confirmation of this observation.

## Abbreviations

Hb: Haemoglobin
SCA: Sickle-cell disease
SCD: Sickle-cell anaemia
